# Quality of life in patients with the first-episode psychosis

**DOI:** 10.1192/j.eurpsy.2021.1420

**Published:** 2021-08-13

**Authors:** A. Usova, A. Khalikova

**Affiliations:** 1 Psychiatry And Medical Psychology, Omsk State Medical University, Оmsk, Russian Federation; 2 Preventive Examination, West Siberian medical center of the Federal medical and biological Agency, Omsk, Russian Federation

**Keywords:** schizophrénia, first-episode psychosis, quality of life

## Abstract

**Introduction:**

The use of a modern biopsychosocial model of care for patients with schizophrenia dictates the need for a dynamic assessment of the quality of life of patients at different stages of the disease to identify targets for treatment and rehabilitation measures. It is especially important to determine the available targets in the early stages of the disease in order to select effective complex therapy and improve the clinical and social prognosis.

**Objectives:**

To determine the socio-biological and clinical factors that have a significant impact on the quality of life of patients with first-episode psychosis.

**Methods:**

The sample consisted of patients diagnosed with schizophrenia (n=58). The following research methods were used: 1) clinical-psychopathological; 2) quality of life assessment; 3) clinical-laboratory and functional research methods; 4) statistical. The QL-100 questionnaire was used as a tool for studying the quality of life. The PANSS scale was used to assess the severity of positive and negative syndromes.

**Results:**

The most significant adverse impact on the quality of life indicators are the absence of family, lack of work, low material wealth, active consumption of psychoactive substances, the predominance of negative symptoms and the presence of concomitant somatic disease in the acute phase.

**Conclusions:**

Early identification of problematic aspects of the quality of life in patients with the first-episode psychosis allows us to conduct effective treatment and rehabilitation measures for these patients.

